# Supplementary material for: Long-term resource addition to a detrital food web yields a pattern of responses more complex than pervasive bottom-up control
Source: PeerJ. 2017 Jul 21;5:e3572. doi: 10.7717/peerj.3572 (PMC5522720; doi:10.7717/peerj.3572)
Supplement: Appendix S1 [file peerj-05-3572-s001.pdf]

Supplemental **Appendix S1**

Analysis of ergosterol in leaf litter

Methods

Two leaves, one each from the top and bottom litter layers, were collected from each plot in August of Year 3. The samples were kept cool and in the dark until brought back to the lab. A disk (ca. 2-cm^2) was cut from each leaf, weighed, and stored in 5 ml of HPLC-grade MeOH in glass scintillation vials (covered with aluminum foil) and refrigerated at 5°C until processed. Ergosterol was extracted by the following procedure: 2 ml of 4% KOH in 95% ethanol were added to the stored samples. The vials were then heated in a water bath (80-85°C) for 30 minutes, removing them after 15 minutes to vortex-mix the contents. Samples were cooled to room temperature and 2 ml of MilliQ H_2O was added, followed by 4 ml of HPLC-grade hexane. Samples were inverted 20x and allowed to settle for 10 minutes. The hexane extract, which contained the ergosterol, was removed with a Pasteur pipet and placed in a new scintillation vial. Hexane (3 ml) was added 2 more times. After each addition the sample was inverted 20x and allowed to settle, and the hexane extract was removed and added to the previous hexane harvest. The hexane harvest was dried under N_2 in a 40°C sand bath and then re-suspended in 2 ml MeOH and stored at -15°C until run in the HPLC.

All analyses were completed on a Varian Inc. HPLC equipped with an Econosil 5µm, C-18 reverse-phase column and guard column (Alltech, Deerfield, IL) in the College of Agriculture, University of Kentucky. HPLC-grade methanol was used as the isocratic mobile phase at a flow rate of 2ml/min at room temperature and a 25-µm injection loop. Detector wavelength was set at 282 nm. This procedure is based on the procedures of Newell et al. (1988) and Suberkropp (1995) modified by Mike Kaufman (*personal communication*). Ergosterol retention time was ca. 7 minutes. Purified ergosterol standard (Sigma Chemical, St. Louis, MO) was diluted in MeOH to concentrations of 0, 1, 2, 5, 10, 25, 50, and 100 ppm. Several dilutions of ergosterol standard were used to derive a linear regression equation for determining ergosterol content of the samples.

Results

Ergosterol content was higher in the resource-subsidized plots, as illustrated in the following figure (* $P_{1,14} < .05$ from 1-way ANOVA after MANOVA ($P_{1,13}$(Wilks' λ) = .004):

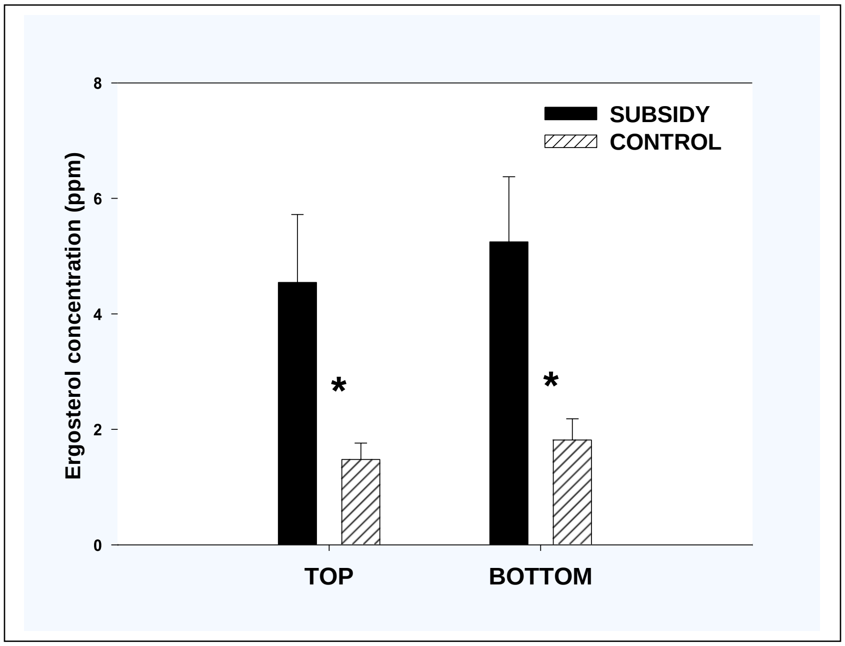

References

Newell, S. Y. et al. 1988. Fundamental procedures for determining ergosterol content of decaying plant-material by liquid-chromatography. – Applied and Environmental Microbiology 54: 1876-1879.

Suberkropp, K. 1995. The influence of nutrients on fugal growth, productivity, and sporulation during leaf breakdown in streams. – Canadian Journal of Botany-Revue Canadienne de Botanique 73: S1361-S1369.
