# Supplementary material for: Long-term resource addition to a detrital food web yields a pattern of responses more complex than pervasive bottom-up control
Source: PeerJ. 2017 Jul 21;5:e3572. doi: 10.7717/peerj.3572 (PMC5522720; doi:10.7717/peerj.3572)
Supplement: Appendix S2 [file peerj-05-3572-s002.pdf]

Supplemental **Appendix S2**

Methodological details of the statistical analyses

Multivariate Analyses

Using the entire data set of all 18 response variables (taxon-sampling method combination) for all treatments, we first used the PRIMER-e / PERMANOVA+ software (Anderson et al. 2008) to calculate a distance matrix for the entire data set using Gower's similarity index [S15 (Legendre and Legendre 2012)] for fourth-root transformed values. Gower's index is appropriate for our data because it is designed to accommodate different types of variables (Kempson, sifting and sticky-trap samples; Fig. 1 in text). We employed S15 because this version of Gower's measure is a symmetrical index that gives equal weight to double zeroes and ++, which is the type of index philosophically appropriate (unlike the commonly used "Bray-Curtis" measure) for analyzing results of a controlled, manipulative field experiment (Legendre and Legendre 2012). We then used permutational multivariate analysis of variance (perMANOVA) to test for interactions with Fencing and Season in the full model (Anderson et al. 2008). Results of these analyses (Supplementary Appendix S3) suggested we could pool Open and Fenced plots before examining how distances between communities in ordination space [PCO (Principal Coordinates Ordination)] changed over time in relation to the Resource treatment for summer and fall samples separately (PCO plots were done with the PRIMER-E / PERMANOVA+ software). We then used perMANOVA to test for a Resource effect in Year 1, and the interaction between (Resource x Year) in Years 2 and 3 for each season.

Univariate analyses

We first plotted univariate vectors on constrained principal coordinates ordinations (CAP in the PRIMER-E / PERMANOVA+ software; Resource and Fencing as constraining factors because they were integral to the experimental design) of those response variables with the highest correlations with the first CAP axis, the one most closely related to the Resource effect (Fig. 3 in text; for all periods except the summer of Year 1, when there was no strong evidence for an effect of Resource on arthropod community structure). We selected vectors based upon two criteria: (1) a simple (i.e. not accounting for co-variation with other variables) Spearman rank correlation with the first CAP axis ≥ .50 or ≤ -.50 ($R^2 \geq 25\%$), or (2) a multiple correlation coefficient (analogous to a univariate partial correlation coefficient) with CAP axis 1 ≥ .35 or ≤ -.35 ($R^2 \geq 12\%$). Results of these correlation analyses with CAP Axis 1 appear in Fig. 3 and Supplementary Appendix S6.

We did not limit our univariate analyses to the examination of vector overlays, but also analyzed each of the 18 response variables separately. To parallel the multivariate analyses, univariate models were fitted separately for summer and fall samples. In addition, a possible interaction with Fencing was evaluated before testing for the simple Resource effect in Year 1 or the Resource x Year interaction for Years 2 and 3. We analyzed the number of individuals sampled, not density (i.e. number per sampling area); thus, the data were count data, which meant that a generalized linear model would be the most appropriate starting point.

Year 1: Even though the data are counts that usually include many zeros, for two response variables a simple linear model was adequate because the distribution had no zeros and was symmetrical, and the residuals were well behaved. However, for all other variables in Year 1 the generalized linear model (GLM) was the starting point. We first tried the Poisson family, then the Negative Binomial. One of these families often produced a model that converged and met model assumptions: Dispersion Index was close to 1.0 (~ .8 to 1.2), residuals were normally distributed, and plots of residuals against predicted values and by treatment were reasonably well behaved. If the data could not be modeled adequately by GLM (Zuur et al. 2009), permutational

univariate analysis of variance (permANOVA; Anderson et al. 2008) was used to model the data; permANOVA was required for 25% (9/36) of the analyses. For Year 1 data we first tested for the Resource x Fencing interaction; if there was evidence of an interaction, models were constructed separately for the Resource effect in Open and Fenced plots. Otherwise, the Resource effect was based upon the simple effect in the full model.

Years 2 and 3: All response variables were first modeled using a Generalized Linear Mixed-effects Model (GLMM) to test for the Resource x Year x Fencing interaction. If there was an interaction with Fencing, the Resource x Year interaction was tested separately for Open and Fenced plots using GLMM. Otherwise, the Resource x Year interaction was based upon the interaction in the full model. If an adequate GLMM model using either the Poisson or Negative Binomial family could not be found, we employed permANOVA. For Years 2 and 3 data, permANOVA was required for 40% (14/36) of the analyses.

The GLM models were run with the "glm()" function in the R "stats" package. The linear model was also implemented with the "glm()" function with family=Gaussian. GLMM Poisson models were run with "glmer()" from the R package "lme4" and GLMM Negative Binomial models were run with "glmer.nb()" in the R package "glmmADMB." Permutational ANOVA's (perMANOVA and permANOVA) were performed with the "adonis" function in the R package "Vegan" (R Core Team 2016). The criterion for accepting 3-way and 2-way interactions involving Resource as being likely was $P \leq .10$. R version 3.3.2 was used for all analyses.

Detailed results of the univariate analyses appear in Supplementary Appendix S6. The pattern of all multivariate and univariate results is depicted in Figure 4.

References

Anderson, M. J. et al. 2008. PERMANOVA+ for PRIMER: Guide to software and statistical methods. – PRIMER-E Ltd, Plymouth, UK.

Legendre, P., and Legendre, L. 2012. Numerical ecology.–Third English Edition edition. Elsevier, Oxford, UK.

R Core Team (2016). R: A language and environment for statistical computing. R Foundation for Statistical Computing, Vienna, Austria. URL https://www.R-project.org/.

Zuur, A. F. et al. 2009. Mixed effects models and extensions in ecology with R.– Springer, New York, NY.
