# Supplementary material for: Long-term resource addition to a detrital food web yields a pattern of responses more complex than pervasive bottom-up control
Source: PeerJ. 2017 Jul 21;5:e3572. doi: 10.7717/peerj.3572 (PMC5522720; doi:10.7717/peerj.3572)
Supplement: Appendix S3 [file peerj-05-3572-s003.pdf]

Supplemental **Appendix S3**

Multivariate analysis with perMANOVA: Interactions of **Resource x Year** with Season

Table S3.1 – Our first analysis was a perMANOVA (> 9900 unique permutations for each statistic) of the distance matrix calculated for the entire data set (all three years) in order to evaluate the evidence for an interaction between Season and the full **Resource x Year** interaction (Year 1 was included because in subsequent analyses the Resource effect was going to be analyzed for Year 1, but separately from Years 2 and 3). There was no interaction between **Resource x Year** and Fencing {*P*[**(Resource x Year)** x Fencing x Season] = .67) and *P*[**(Resource x Year)** x Fencing] = .22}. Thus, these SS, along with all other interaction SS's for which *P* > .20, were combined with the error SS. There is evidence of an interaction between **(Resource x Year)** and Season (*P* = .037), which means that analyses of the **Resource x Year** interaction, and simple Resource effects for Year 1, should be done separately for summer and fall.

**Source**	**df**	**MS**	**Pseudo-*F***	***P***
Resource	1	2079.1	10.64	< .001
Year	2	2614.3	13.37	< .001
Fencing	1	618.5	3.16	.006
Season	1	5097.3	26.08	< .001
**Resource x Year**	2	510.8	2.61	.003
Resource x Fencing	1	523.9	2.68	.017
Resource x Season	1	569.1	2.91	.009
Year x Season	2	2914.8	14.91	< .001
**(Resource x Year)** x Season	2	367.9	1.88	.039
Pooled	106	195.5		
Total	119			

Table S3.2. Results of perMANOVA (> 9900 unique permutations for each statistic) restricted to Years 2 and 3, when biweekly rates of detrital supplementation were equal, and were ~4x higher than Year 1 (see text for details). This analysis directly tests whether the multivariate impact of the Resource treatment differed over time. Residual SS have been pooled with the SS for the 4-way interaction that included Fencing (*P* > .20). The effect of Resource supplementation on community structure differed between Years 2 and 3 [*P***(Resource x Year)**] = .012]. The evidence for an interaction with Season was weaker (*P* = .15) than for the analysis of the entire 3-year data set, suggesting that the **Resource x Year** interaction for Years 2 and 3 was similar each season; however, separate perMANOVA's by Season in Supplementary Appendix S4 contradict this inference [*P*(R x Y) = 0.22 for summer, *P*(R x Y) = .006 for fall) . The biological rationale for analyzing results separately by Season, along with the evidence for an interaction with Season in Table S3.1 and the suggestion (though weak) of an interaction in Table S3.2, led to all subsequent analyses being done separately by Season.

**Source**	**df**	**MS**	**Pseudo-*F***	***P***
Resource	1	1923.2	9.21	<.001
Year	2	3036.4	14.53	<.001
Fencing	1	472.9	2.26	.043
Season	1	8506.3	40.72	<.001
**Resource x Year**	1	628.4	3.01	.012
Resource x Fencing	1	412.5	1.97	.069
Resource x Season	1	867.6	4.15	.002
Year x Season	1	572.65	2.74	.024
**(Resource x Year)** x Season	1	333.51	1.61	.15
Pooled	70	207.1		
Total	79			
