# Supplementary material for: Long-term resource addition to a detrital food web yields a pattern of responses more complex than pervasive bottom-up control
Source: PeerJ. 2017 Jul 21;5:e3572. doi: 10.7717/peerj.3572 (PMC5522720; doi:10.7717/peerj.3572)
Supplement: Appendix S4 [file peerj-05-3572-s004.pdf]

Supplemental **Appendix S4**

Multivariate analyses (perMANOVA) of the **Resource** effect for each Year/Season combination

Table S4.1 – perMANOVA's of the **Resource x Year** interactions by (A) SUMMER and (B) FALL (paralleling the full perMANOVA's of Supplementary Appendix S3 for all years, and only Years 1 and 2), followed by separate perMANOVA's of **Resource** and **Fencing** for each Year for summer and fall separately. For all analyses, interaction SS were pooled with error (residuals) SS if $P > .20$. Re = Resource, Fe = Fencing, Res = Residuals. Unique permutations > 9900 for all tests.

(A) SUMMER

All three years: P[Pseudo-$F_{1,53}$ (Resource x Year)] = .04

<u>Years 2 and 3</u>: P[Pseudo-$F_{1,35}$ (Resource x Year)] = .22

<u>YEAR 1</u>

Source	df	SS	MS	Pseudo-*F*	*P*
Re	1	205	205.2	1.681	.15
Fe	1	314	314.3	2.575	.031
Re x Fe	1	239	239.3	1.960	.087
Res	16	1953	122.0		
Total	19	2712			

<u>YEAR 2</u>

Reduced perMANOVA [*P*(Re x Fe) = *.52]*

Source	df	SS	MS	Pseudo-*F*	*P*
Re	1	526	526.0	3.080	.005
Fe	1	350	349.9	2.049	.058
Pooled	17	2903	170.8		
Total	19	3779			

<u>YEAR 3</u>

Reduced perMANOVA [*P*(Re x Fe) = *.47]*

Source	df	SS	MS	Pseudo-*F*	*P*
Re	1	720	719.6	3.217	.012
Fe	1	337	337.0	1.507	.18
Pooled	17	3802	223.7		
Total	19	4859			

(B) FALL

All three years: *P*[Pseudo-$F_{1,53}$ (Resource x Year)] = .003

<u>Years 2 and 3</u>: *P*[Pseudo-$F_{1,35}$ (Resource x Year)] = .006

<u>YEAR 1</u>

Reduced perMANOVA [P(Re x Fe) = .22]

Source	df	SS	MS	Pseudo-*F*	*P*
Re	1	448	447.7	2.023	.035
Fe	1	210	210.3	0.950	.50
Pooled	17	3763	221.3		
Total	19	4421			

<u>YEAR 2</u>

Reduced perMANOVA[P(Re x Fe) = .24]

Source	df	SS	MS	Pseudo-*F*	*P*
Re	1	1764	1763.5	8.219	.001
Fe	1	305	304.7	1.420	.19
Pooled	17	3648	214.6		
Total	19	5716			

<u>YEAR 3</u>

Reduced perMANOVA [P(Re x Fe) = .41]

Source	df	SS	MS	Pseudo-*F*	*P*
Re	1	744	743.6	3.285	.004
Fe	1	191	191.5	0.846	.55
Pooled	17	3848	226.4		
Total	19	4783			
