# Supplementary material for: Long-term resource addition to a detrital food web yields a pattern of responses more complex than pervasive bottom-up control
Source: PeerJ. 2017 Jul 21;5:e3572. doi: 10.7717/peerj.3572 (PMC5522720; doi:10.7717/peerj.3572)

Supplemental **Appendix S5**

Univariate Plots: Patterns of change over time of the individual response variables, Figures S5.1 to S5.18

Here we present plots over time of mean abundances ± SE for Supplemented and Ambient treatments for all 18 response variables. Data are presented first for open and fenced plots pooled, followed by patterns for the open and fenced plots considered separately. Summer patterns are presented first, then fall patterns. Response variables are presented in the order of declining overall abundance (Fig. 1 of the text).

Results of statistical modelling of these patterns appear in Supplemental Appendix S6.

KEY:

● Supplemented

○ Ambient

Fig. S5.1 - **Hypogastruridae** (Hyp)

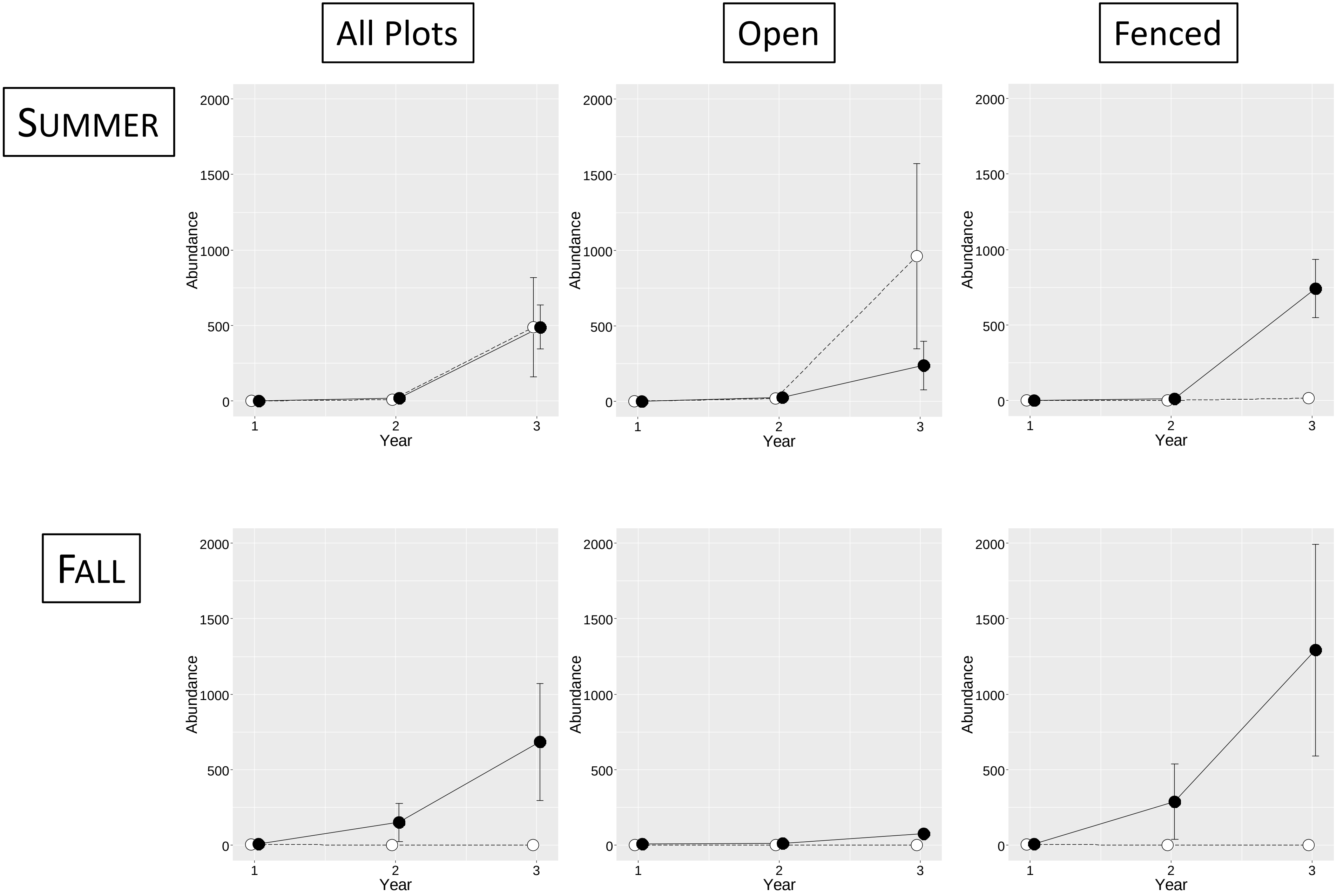

Fig. S5.2 - **Onychuridae** (Ony)

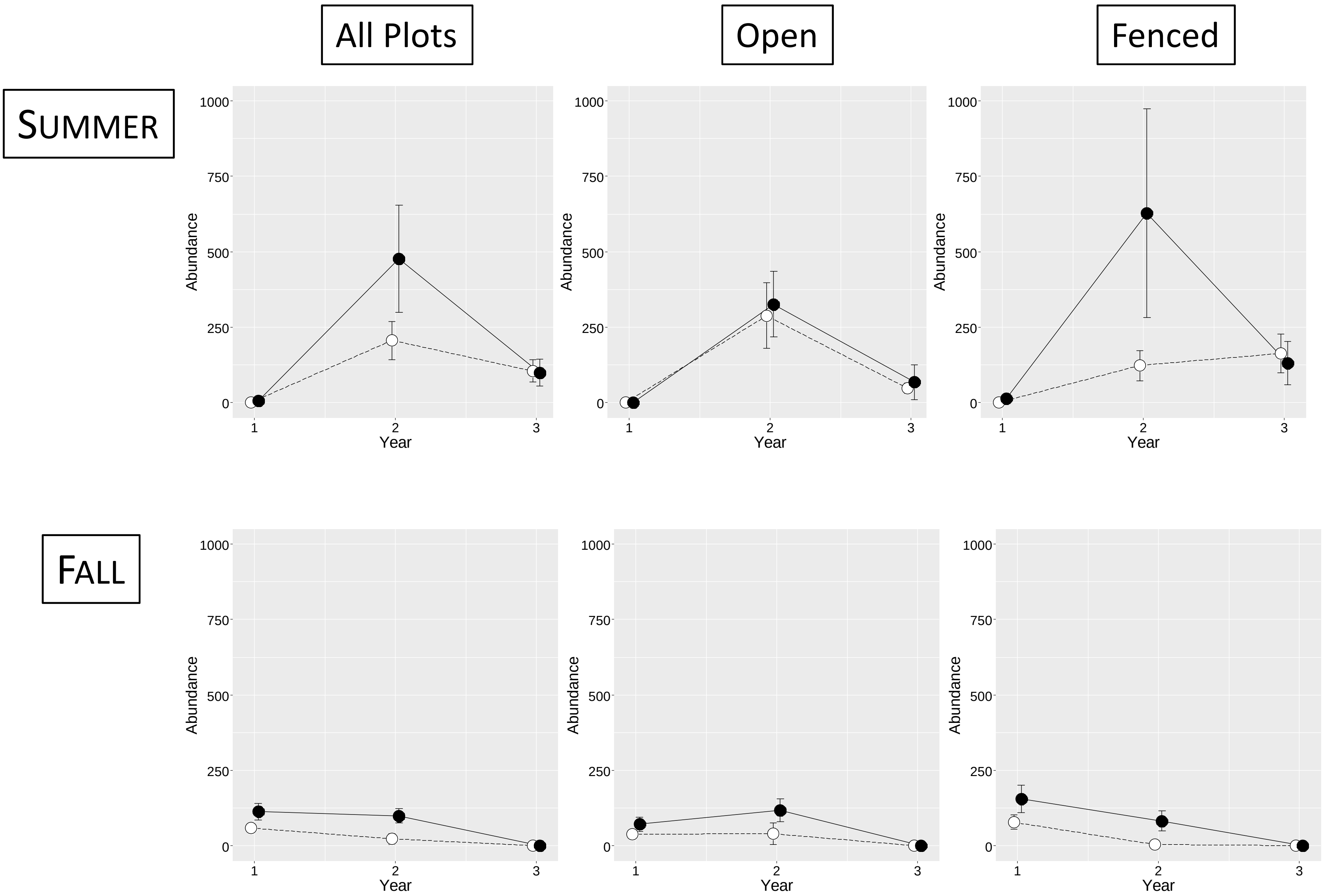

Fig. S5.3 - **Entomobryidae** (Ent)

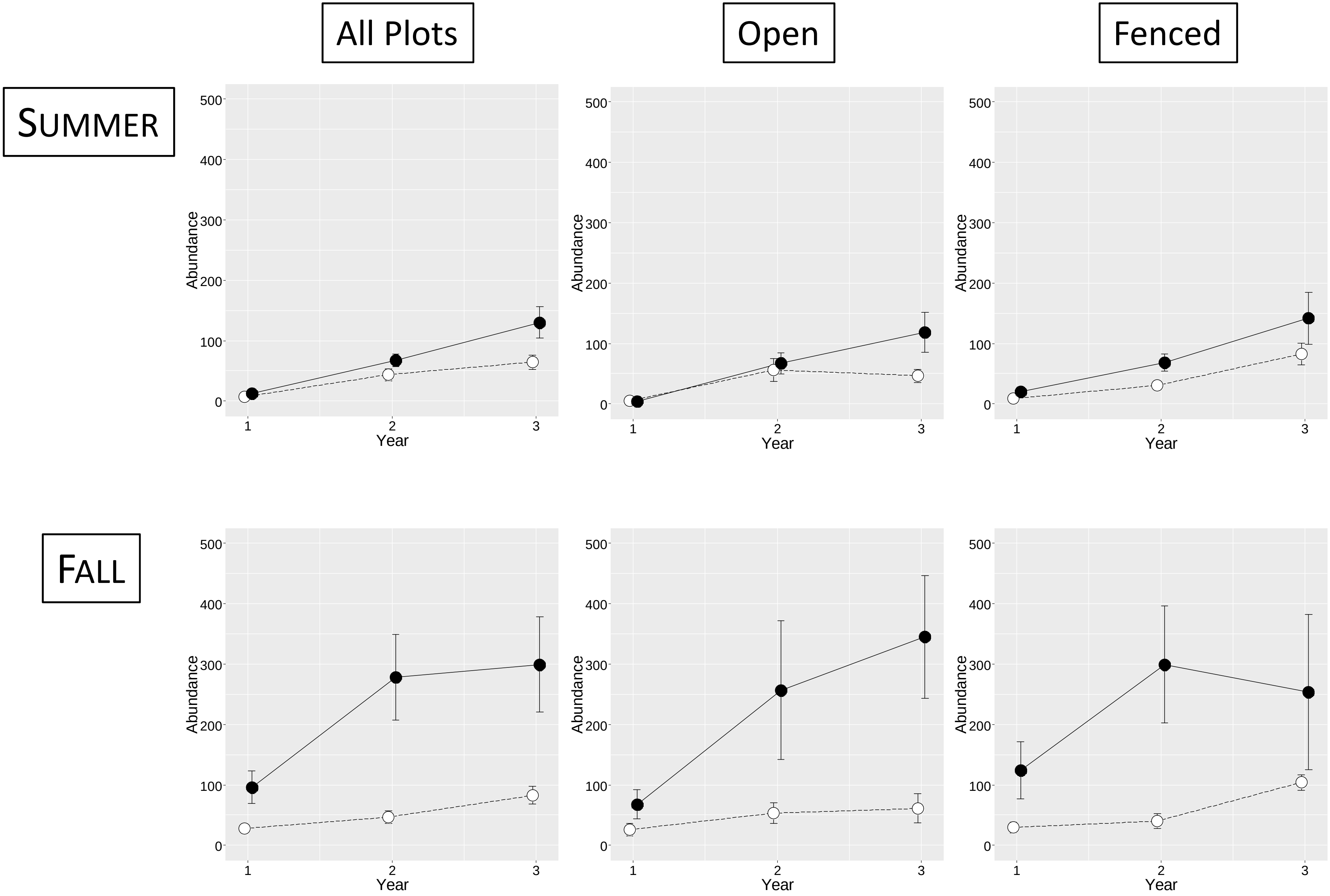

Fig. S5.4 - **Isotomidae** (Iso)

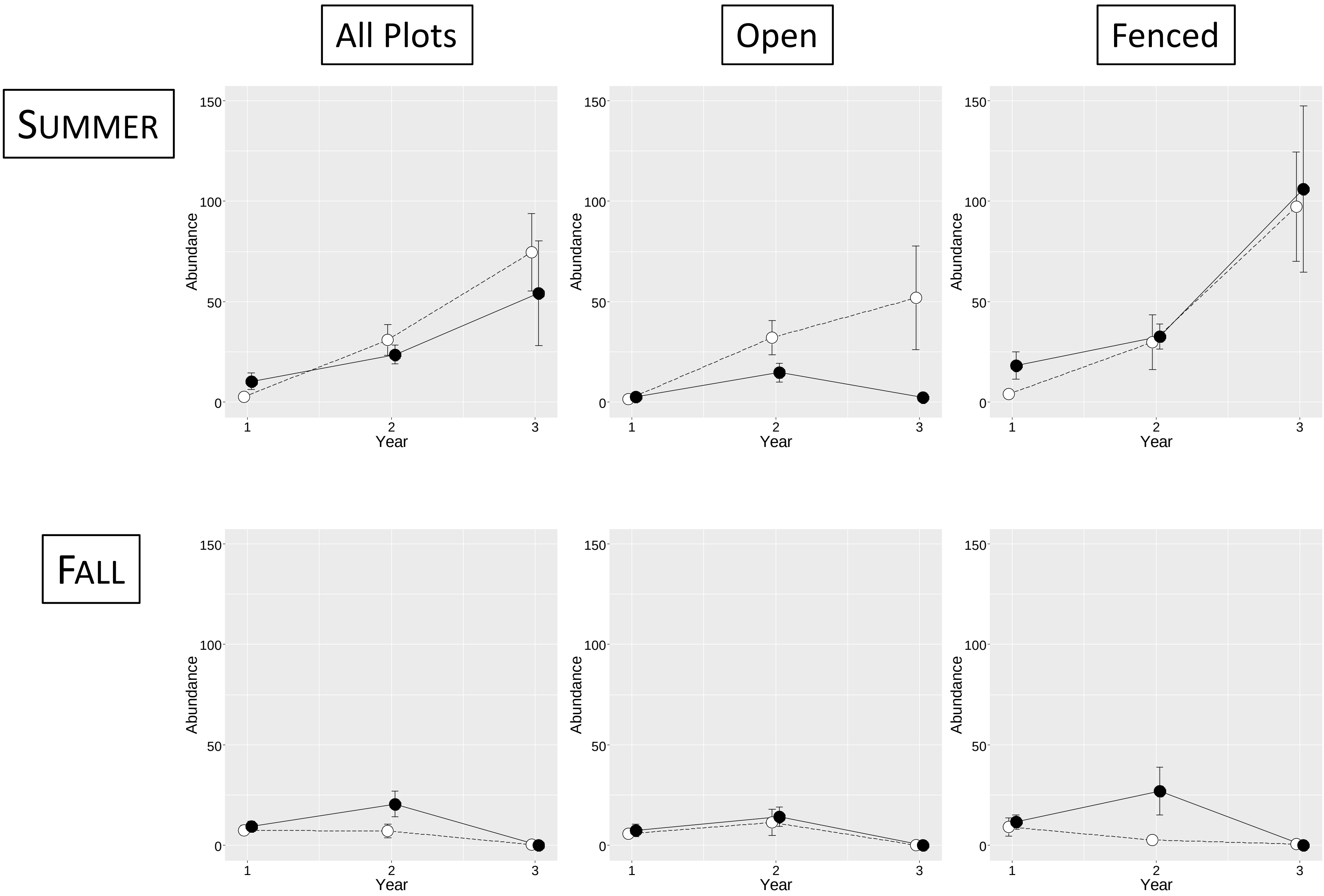

Fig. S5.5 - **Tomoceridae** (Tom)

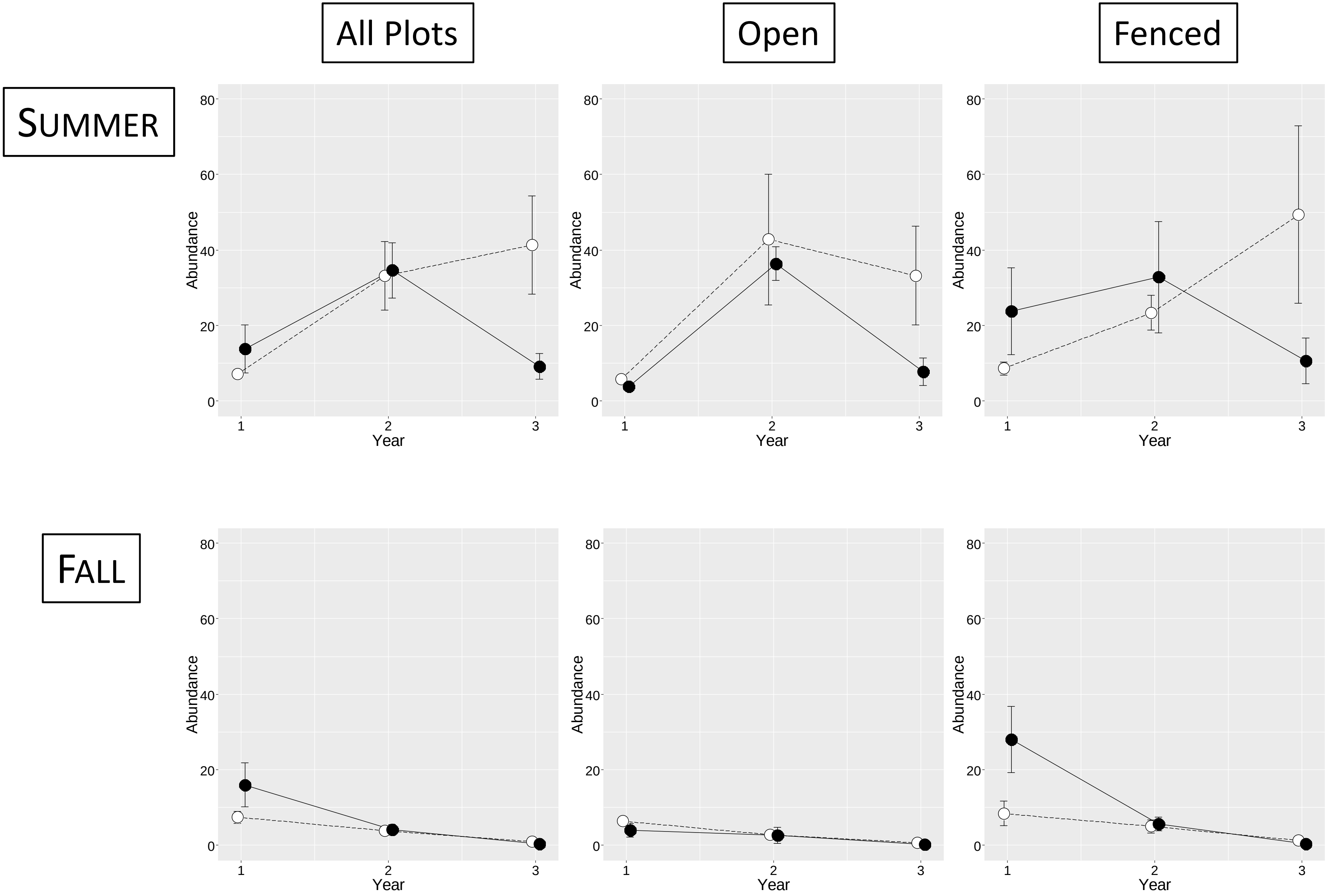

Fig. S5.6 - **Sminthuridae** (Smi)

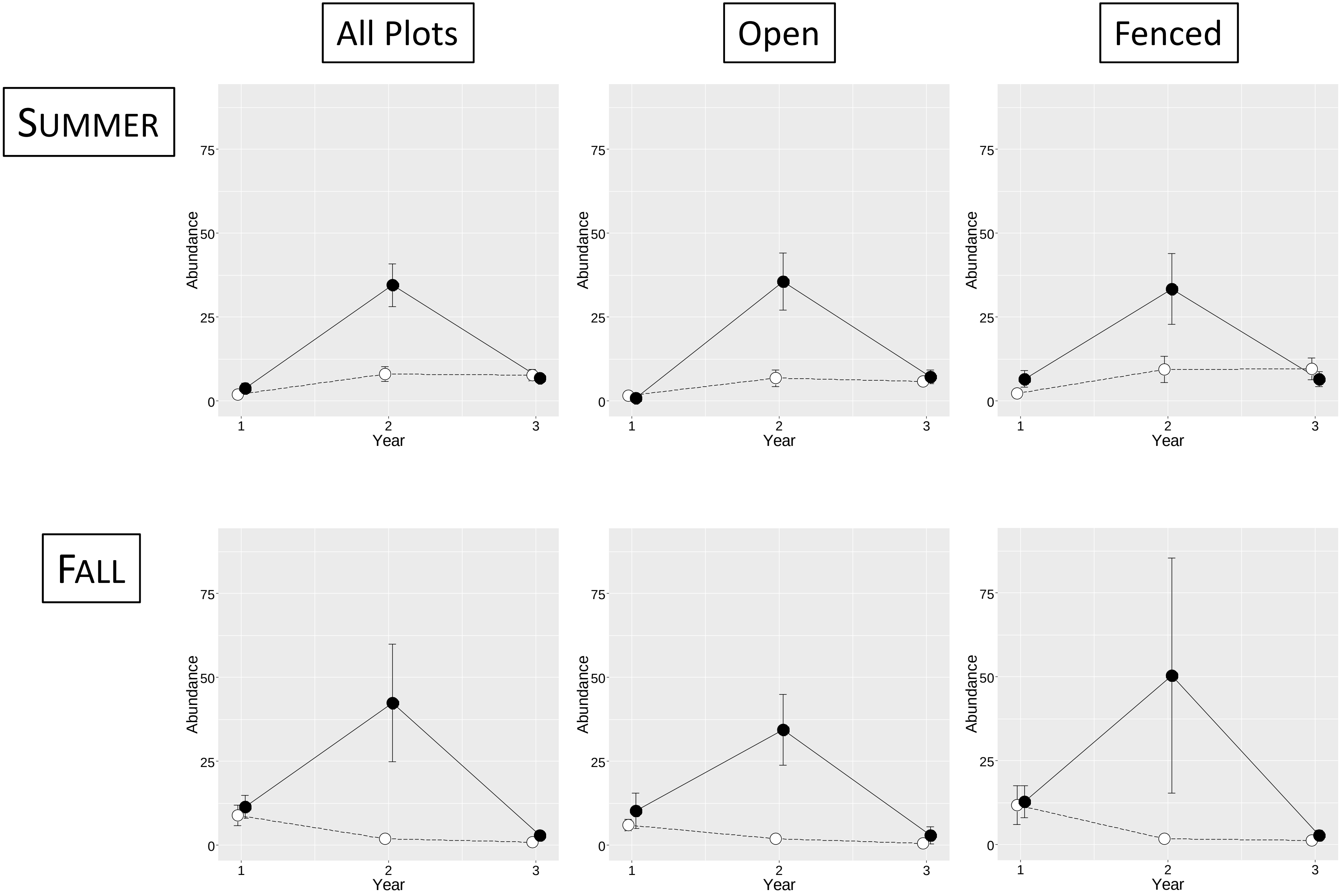

Fig. S5.7 - **Thysanoptera** (Thy)

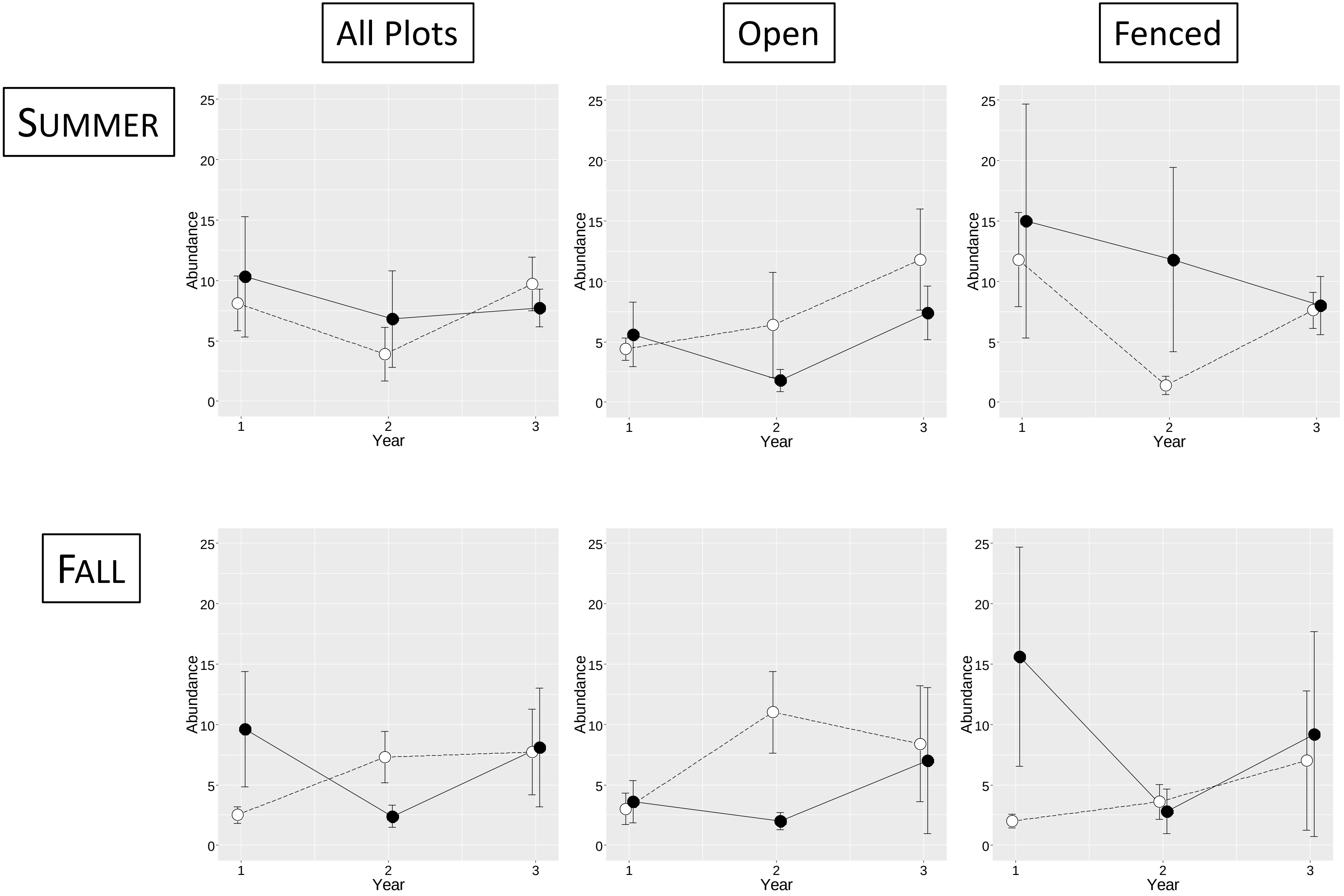

Fig. S5.8 - **Diptera Adults** (TrpDip)

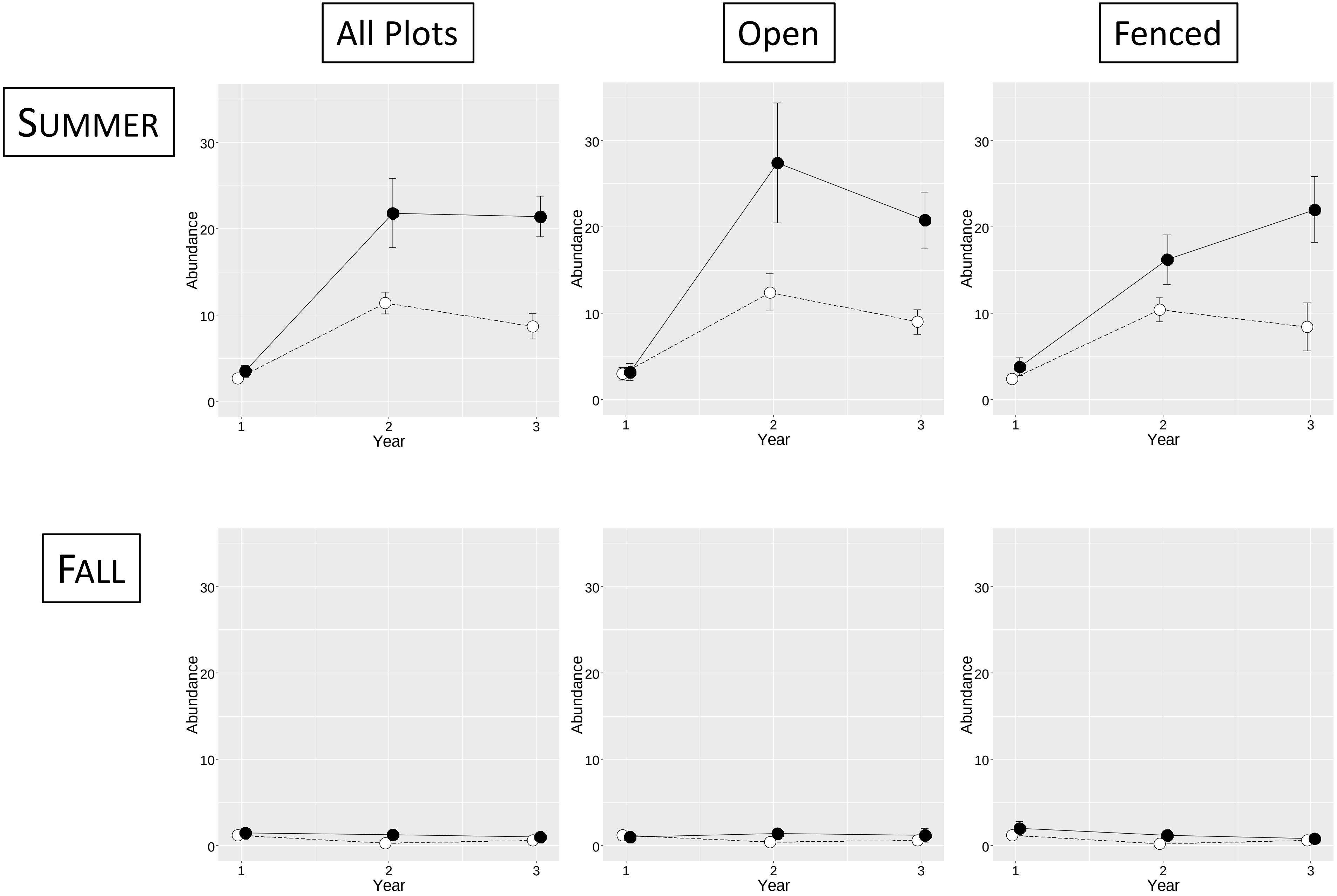

Fig. S5.9 - **Lepidoptera Larvae** (Llep)

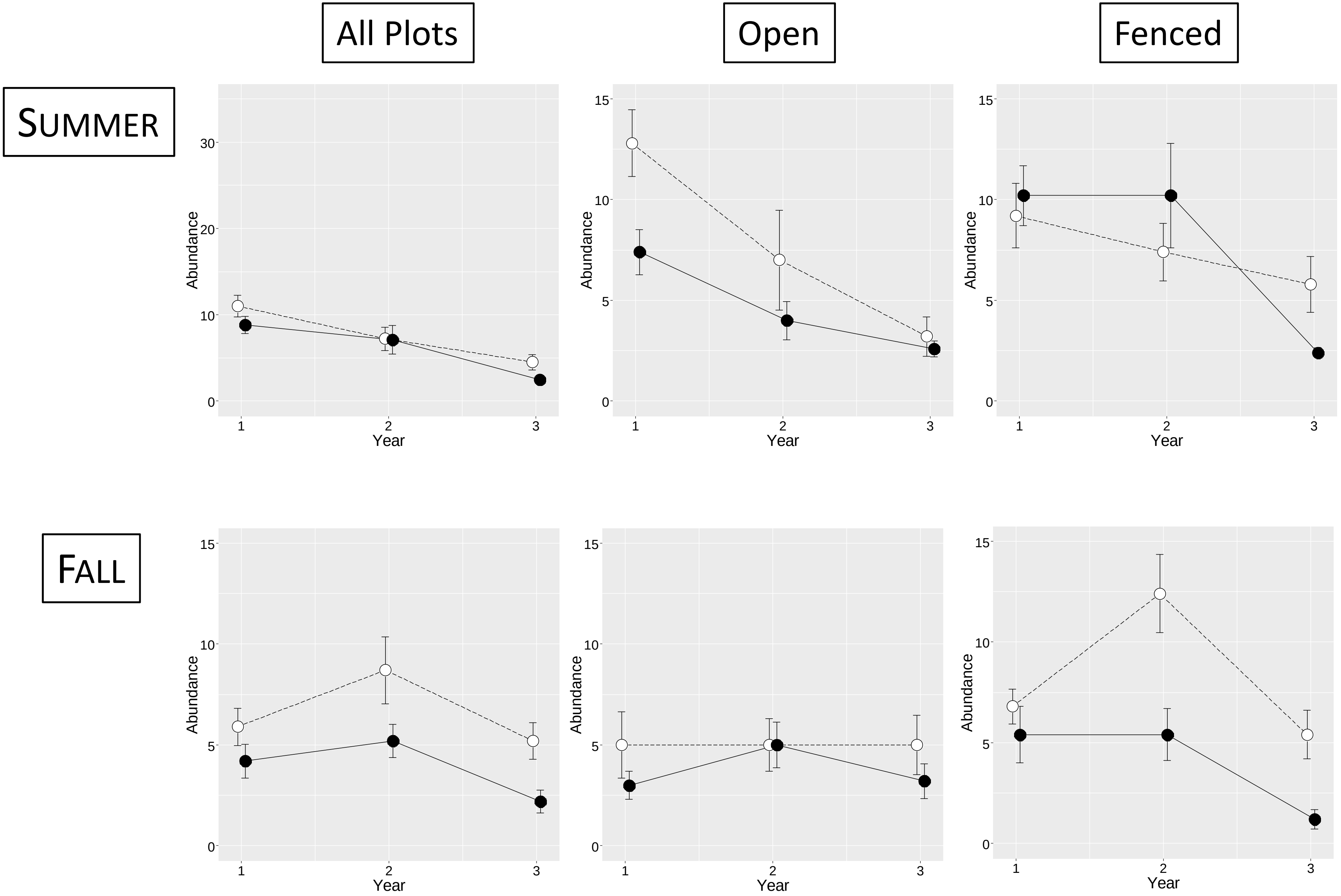

Fig. S5.10 - **Diptera Larvae** (Ldip)

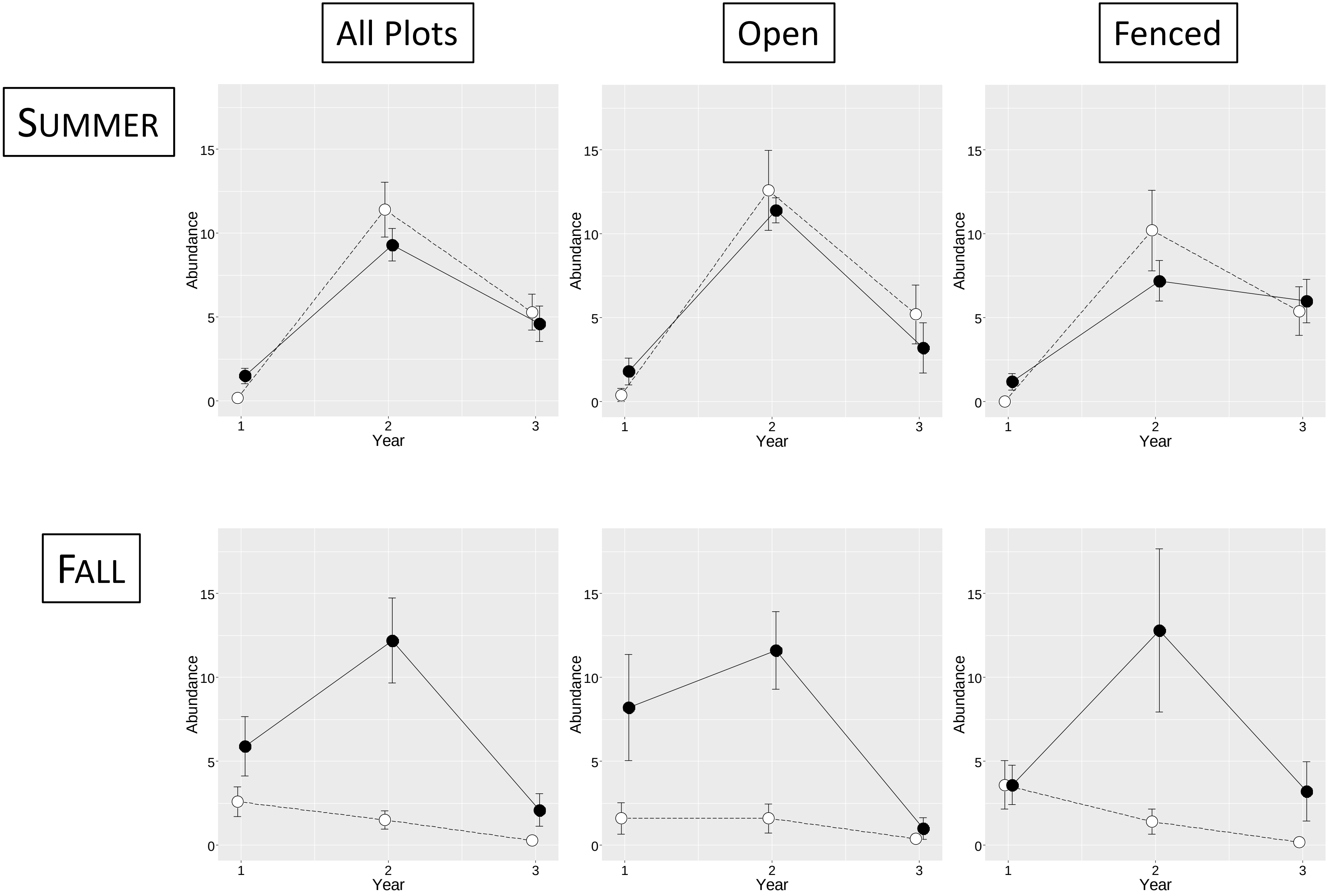

Fig. S5.11 - **Diptera Adults** (Adip)

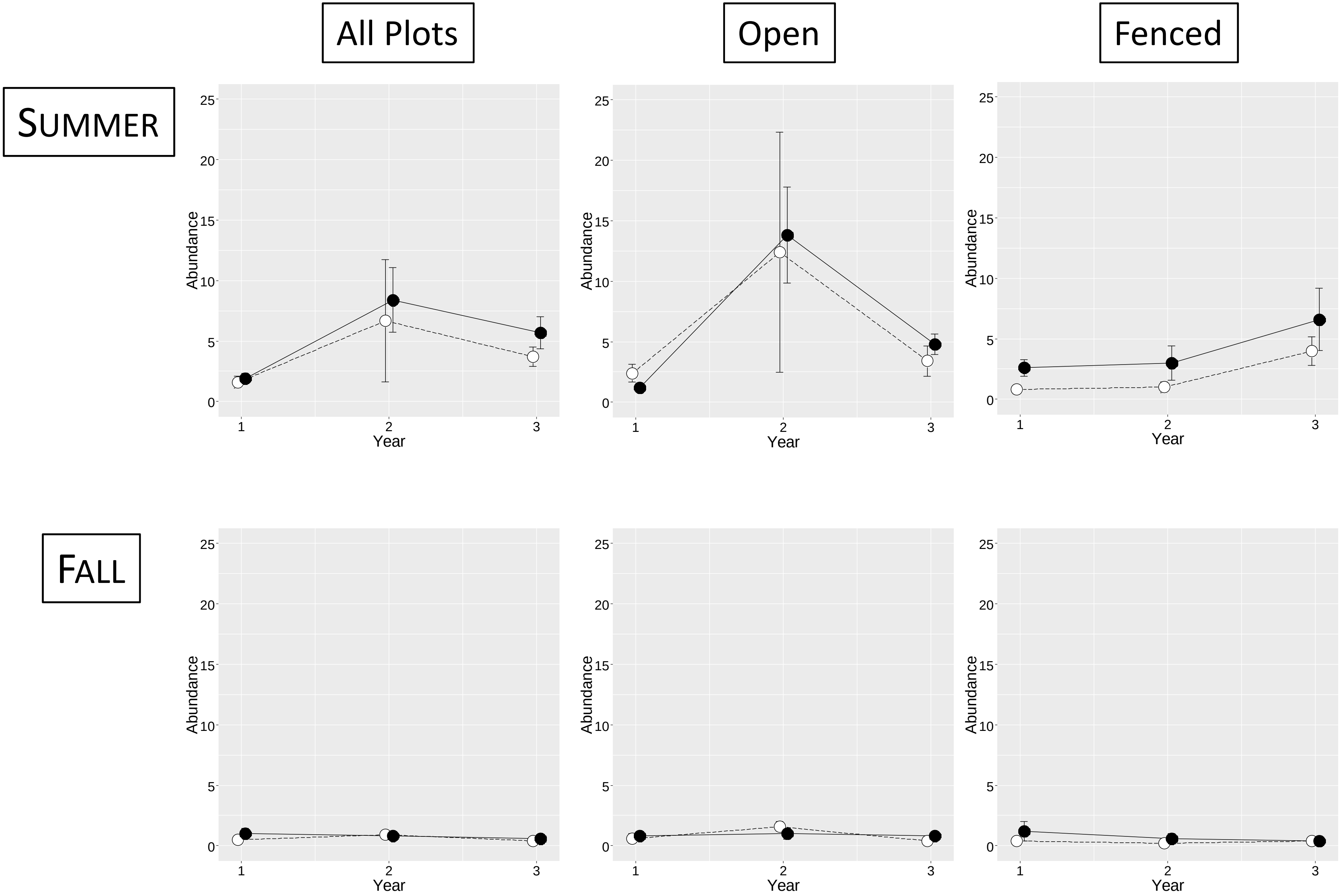

Fig. S5.12 - **Coleoptera Larvae** (Lcol)

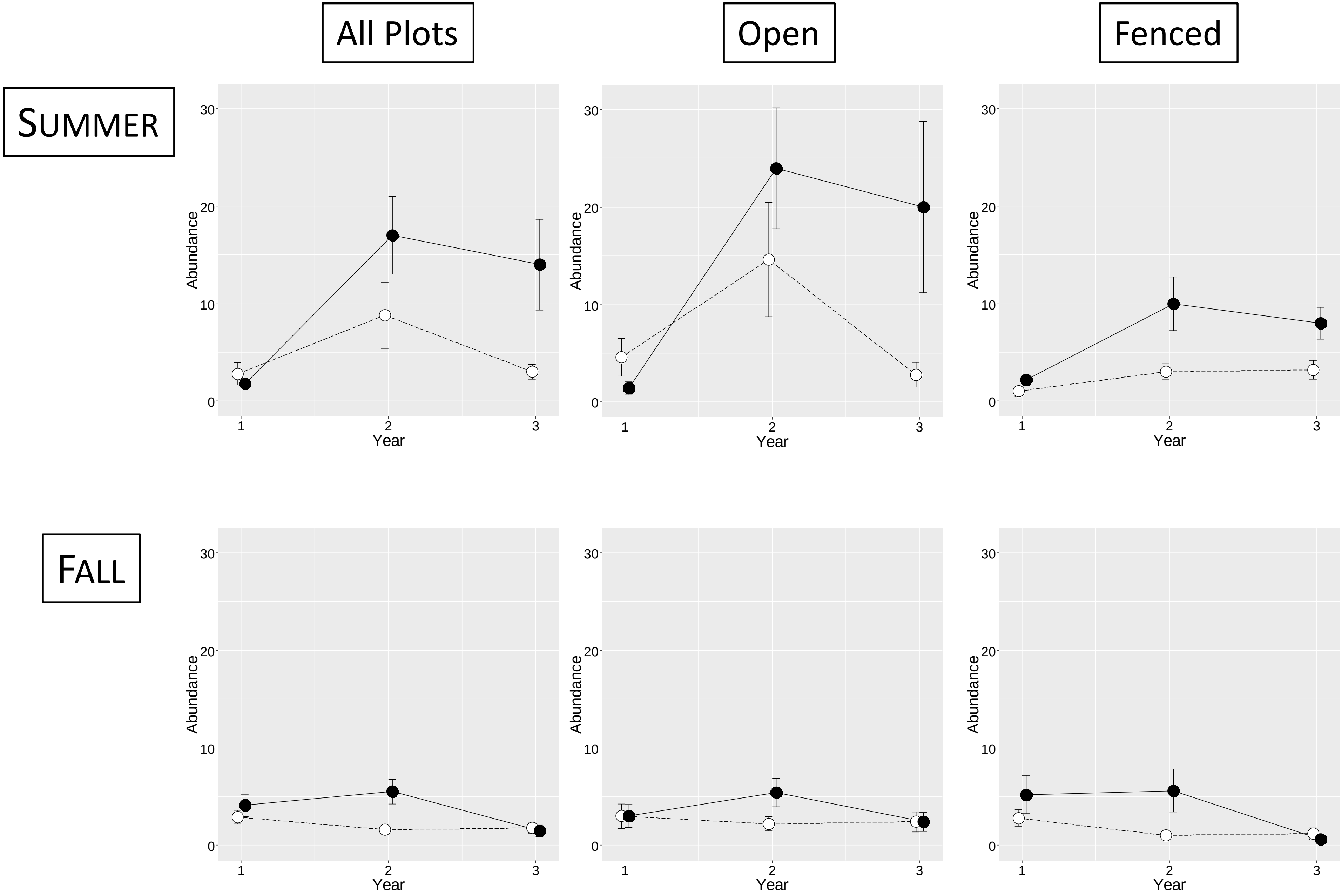

Fig. S5.13 - **Coleoptera Adults** (Acol)

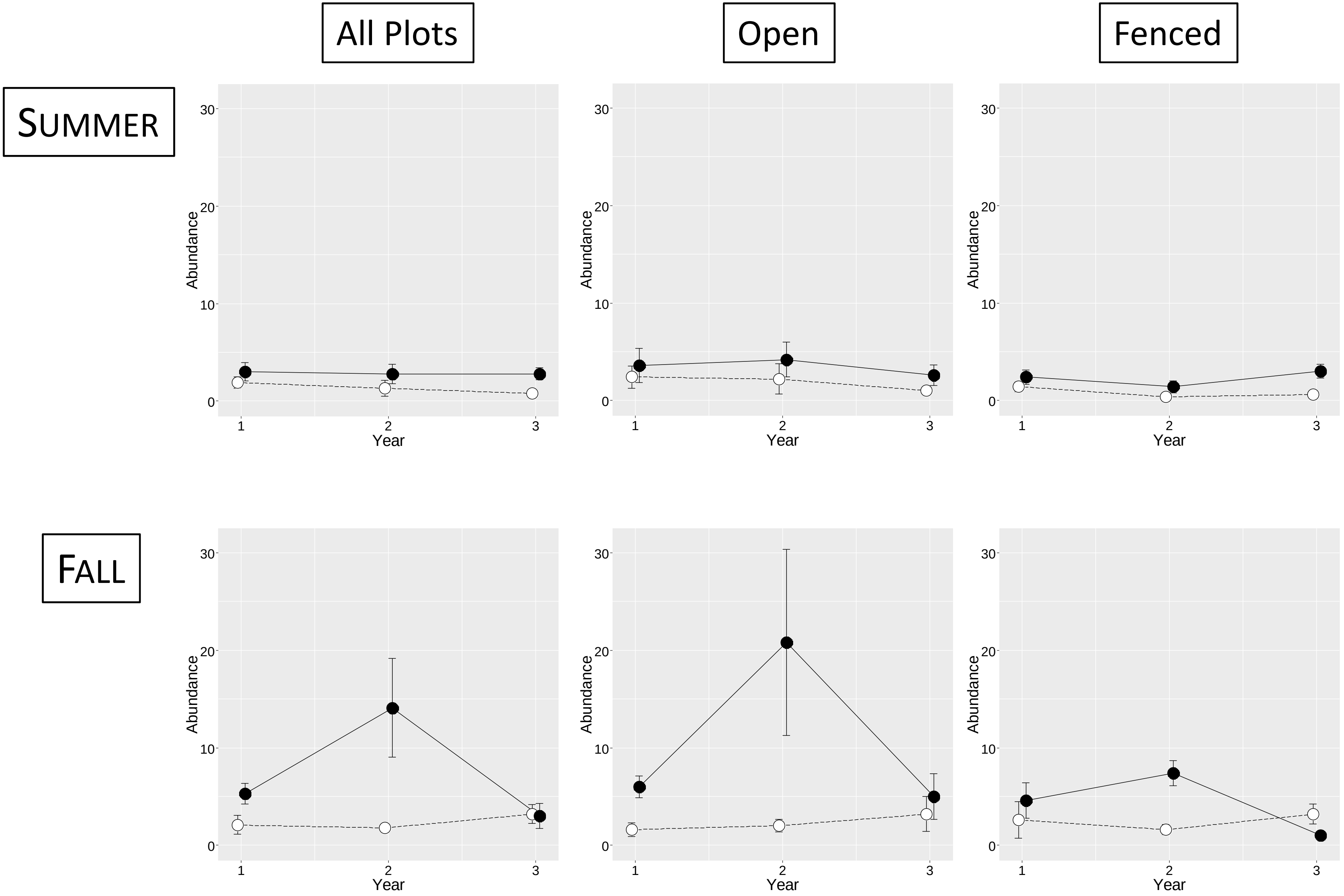

Fig. S5.14 - **Cursorial Spiders** (Cur)

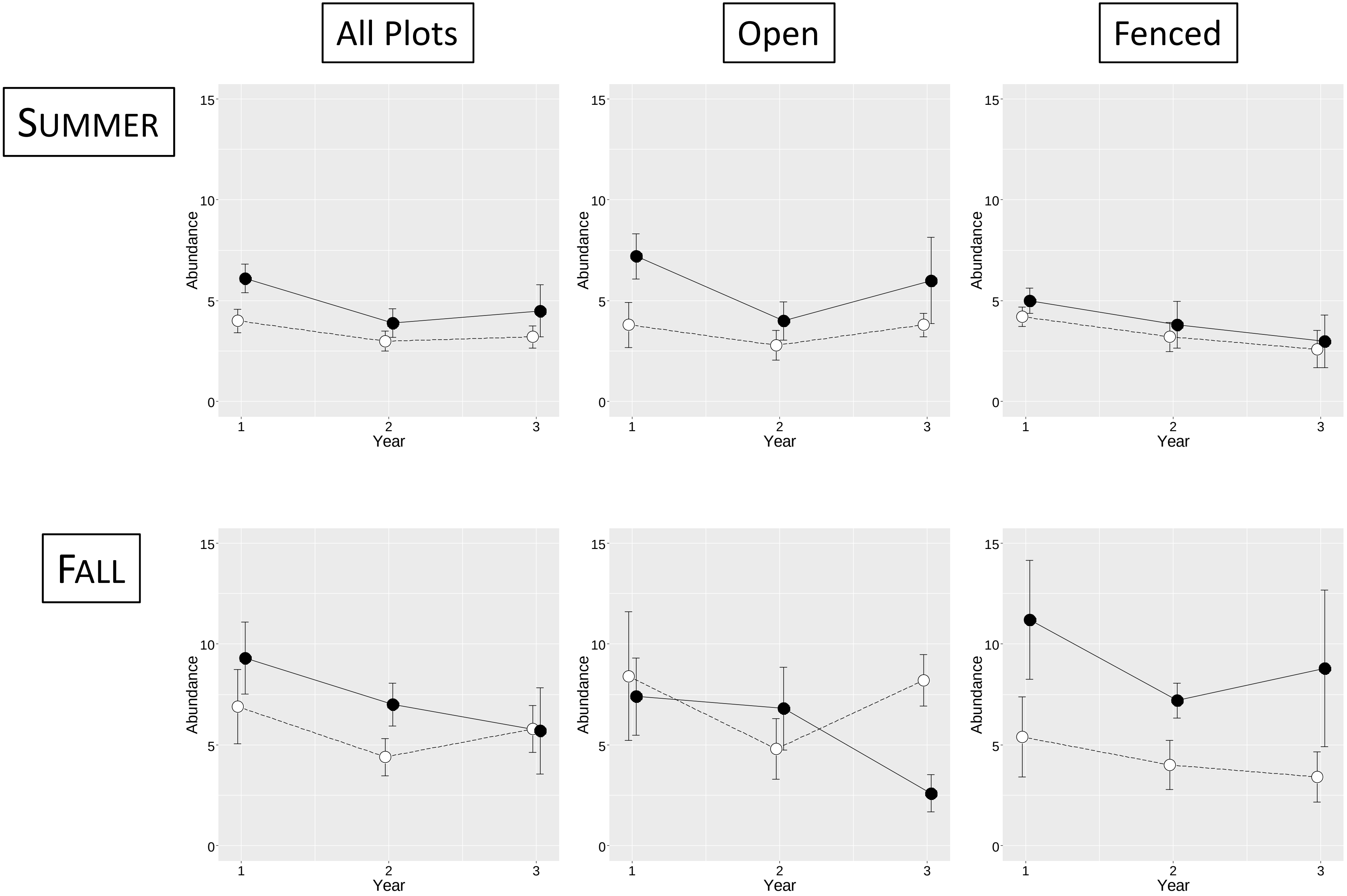

Fig. S5.15 - **Total Spiders** (Ara)

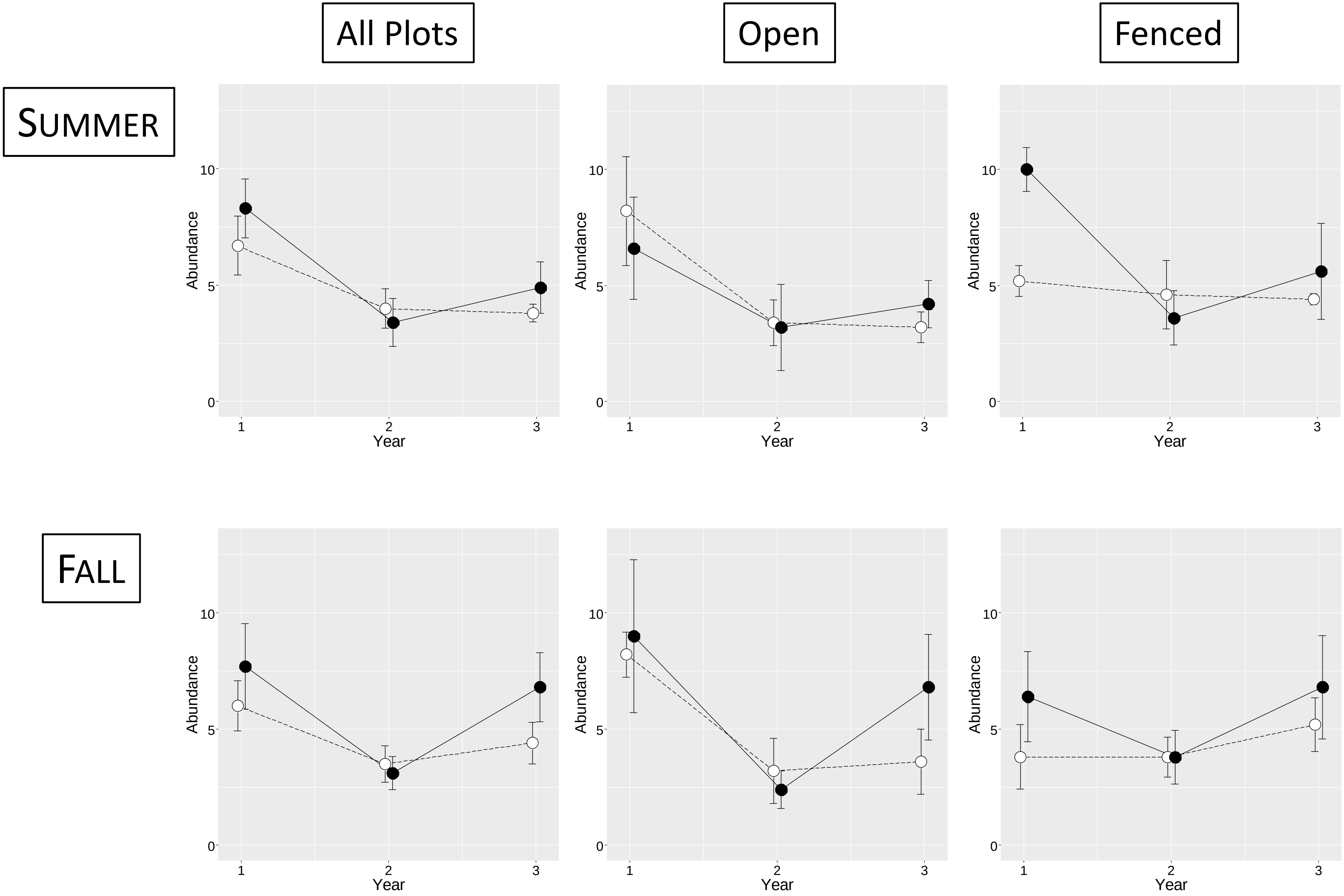

Fig. S5.16 - **Pseudoscorpiones** (Pse)

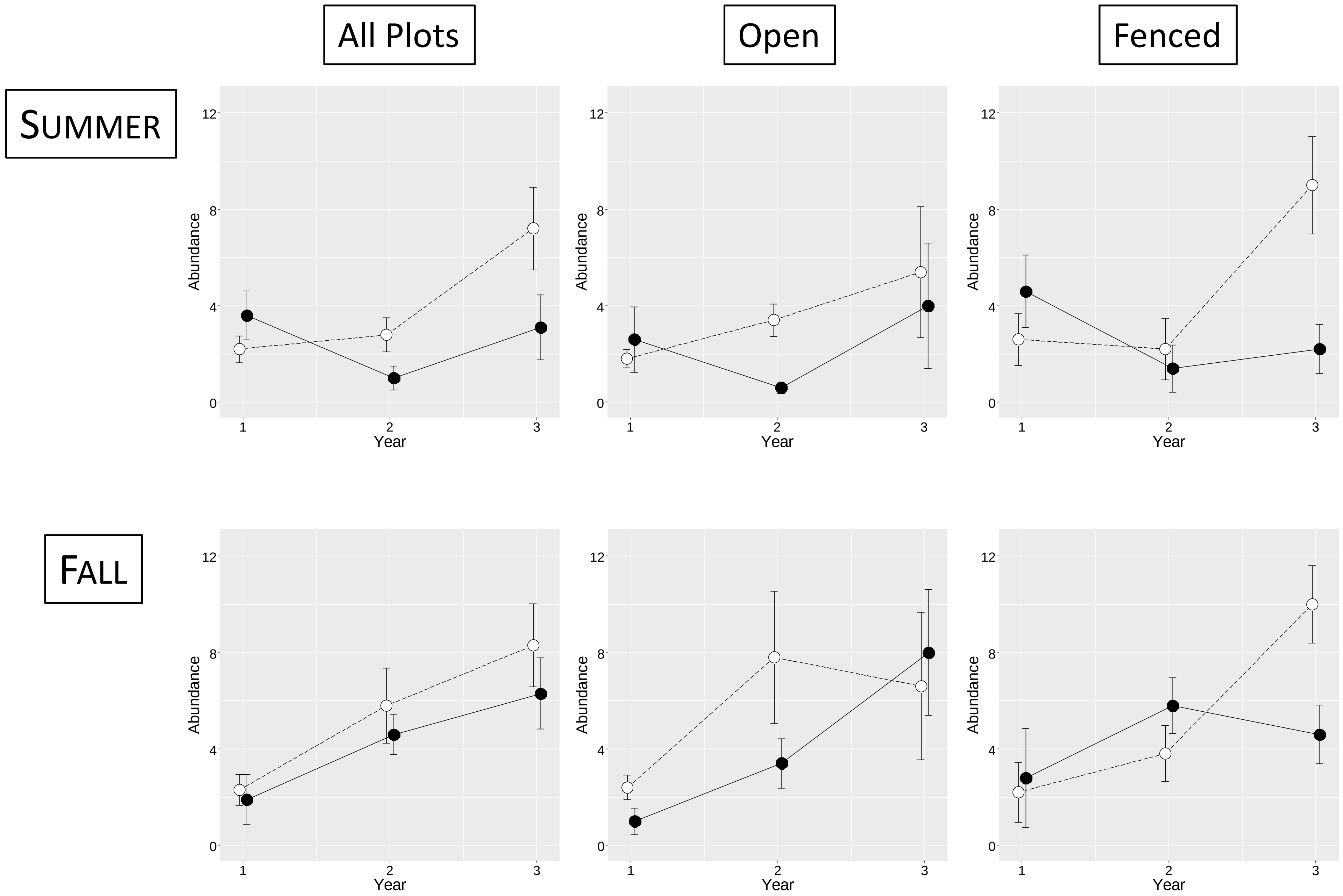

Fig. S5.17 - **Web Spiders** (Web)

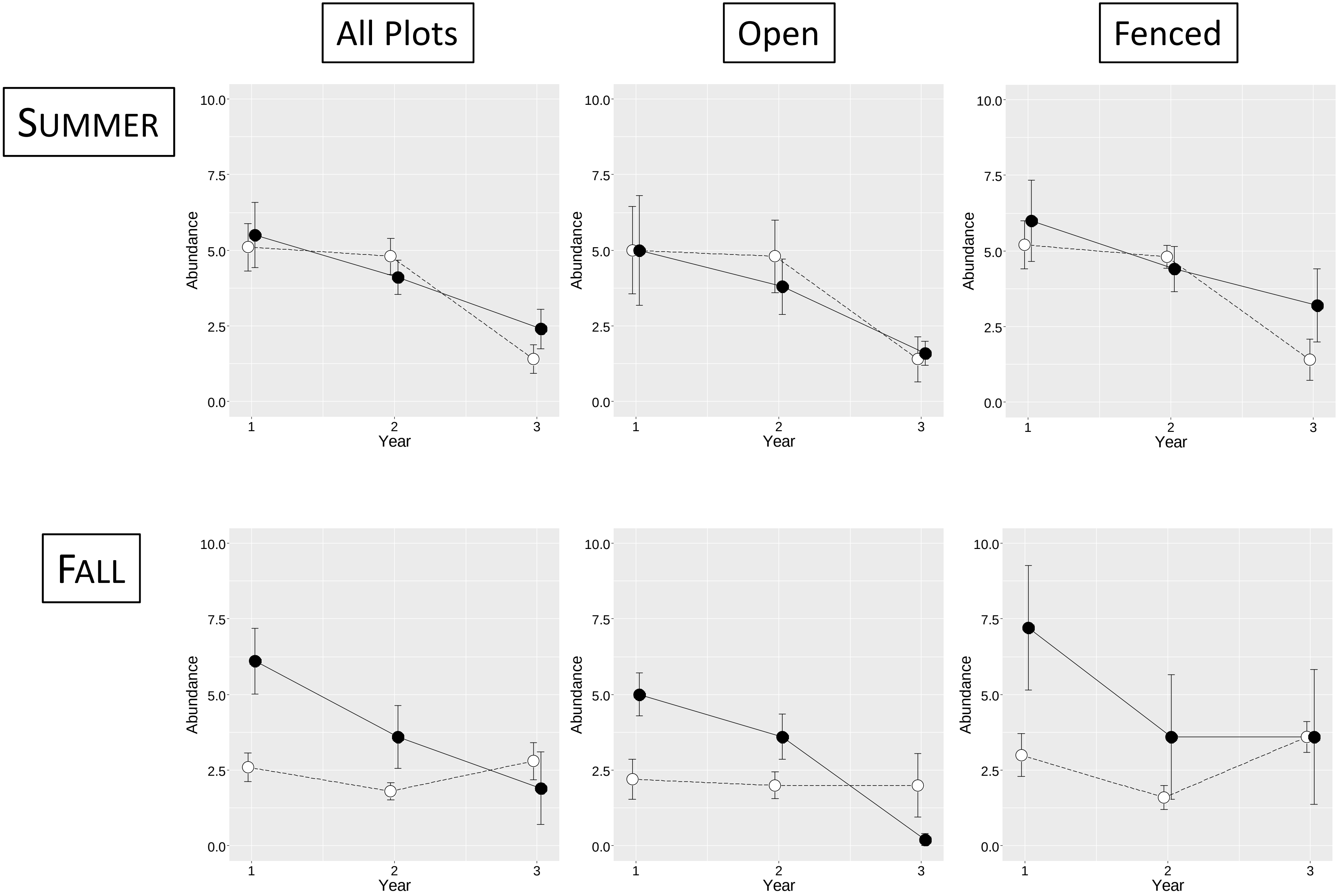

Fig. S5.18 - **Chilopoda** (Chi)

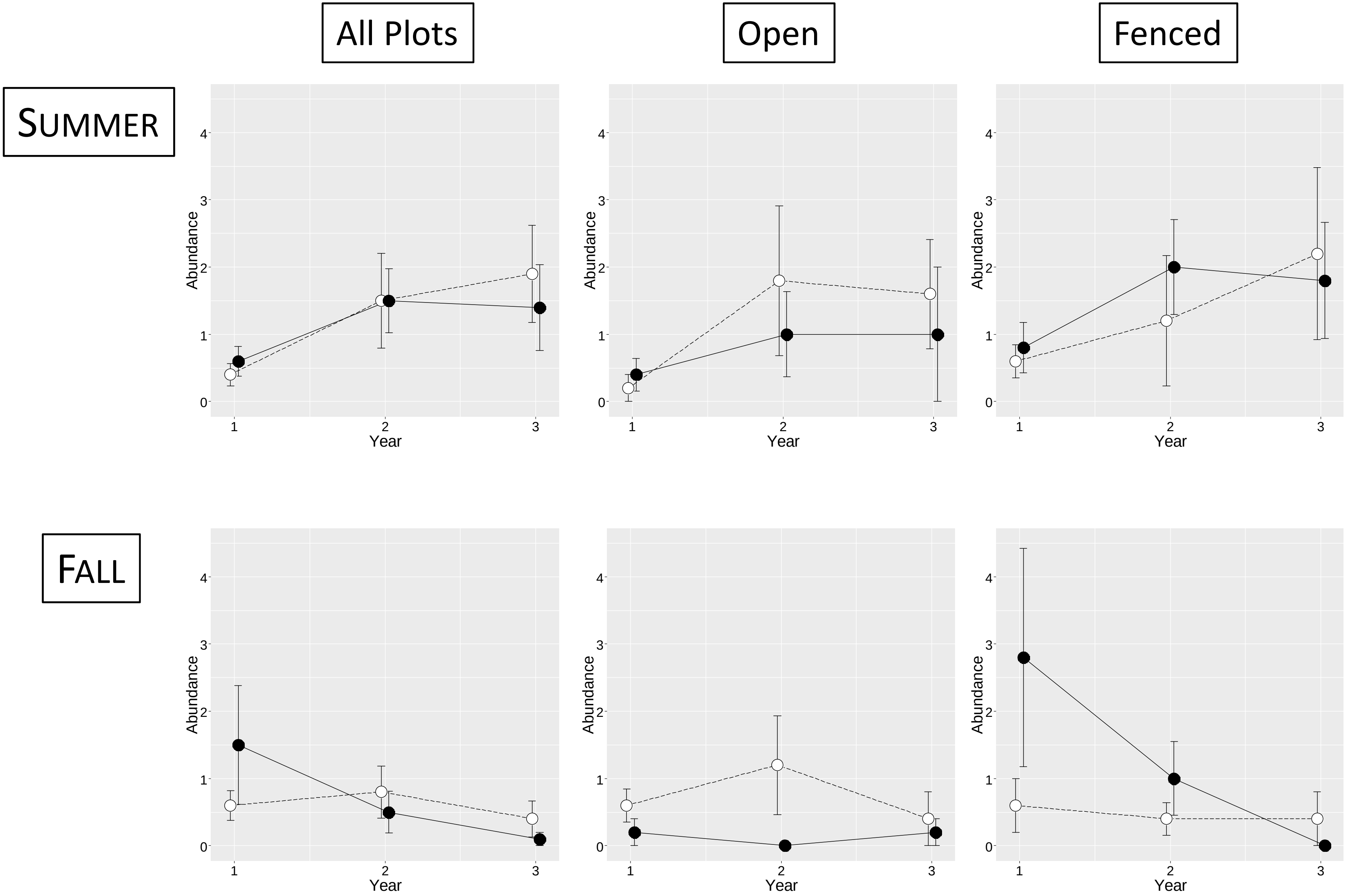

Supplement: Appendix S5 [file peerj-05-3572-s005.pdf]
