# Supplementary material for: Long-term resource addition to a detrital food web yields a pattern of responses more complex than pervasive bottom-up control
Source: PeerJ. 2017 Jul 21;5:e3572. doi: 10.7717/peerj.3572 (PMC5522720; doi:10.7717/peerj.3572)
Supplement: Appendix S6 [file peerj-05-3572-s006.pdf]

Supplemental **Appendix S6:**

Results of univariate analyses and patterns of CAP vector overlays, Tables S6.1 to S6.4.

KEY to Tables S6.1 to S6.4:

Multivariate: Entry indicates the presence of a vector in one of the CAP ordination plots in Figure 3.

Simple	= Spearman rank correlation
Multiple	= Multiple (partial correlation coefficient)
**x**	**=** Vector correlated with a CAP axis, but no correlation with the Resource treatment (Summer, Year 1)
**+**	**=** Positive correlation between response variable and the Resource treatment
–	**=** Negative correlation between response variable and the Resource treatment
Y2, Y3	= Years 2, Years 3

Univariate: Numerical entry is the *P* value of the term (interaction or main effect) from the statistical model. *P* values ≤ .10 are highlighted in **bold.**

Model		= Statistical model used to model the data, all run in R [Y1: in separate column; Y2+Y3, in () with *P* for 3-way interaction]
	L	= Linear Model
	L/Tr	= Linear Model on transformed data
	P	= Poisson Family for Generalized Linear Model (GLM; Y1) or Generalized Linear Mixed-effects Model (GLMM; Y2+Y3)
	NB	= Negative Binomial Family for GLM or GLMM
	Perm	= PermANOVA (Permutational Univariate Analysis of Variance)
R		= R (Ambient or Supplemented)
Y		= Year (Y1, Y2, Y3)
F		= Fencing (Open or Fenced)
R x Y x F		= Three-way interaction; tested first
R x Y		= Two-way interaction if no three-way interaction; or separately for Open and Fenced plots
R		= Effect of Resource addition if no R x Y interaction; separately for Year if R x Y interaction

Notes: Comments on analyses with poorly behaved statistical models and/or analyses that are somewhat at odds with the patterns depicted in one of the univariate plots in Figures S5.1 to S5.18 in Supplementary Appendix S5

Table S6.1: SUMMER, Year 1

Taxon (response variable)	Multivariate		Univariate							
			ALL PLOTS			Open		Fenced		
	Spearman	Multiple	R x F	Model	R	Model	R	Model	R	**Notes**
Fungivores/Detritivores										
Hypogastruridae			.73	Perm	1.00					
Onychuridae			.64	Perm	.89					
Entomobryidae			.13	NB	**.037**					
Isotomidae	**x**	**x**	.39	NB	**.027**					
Tomoceridae			.070	Perm	.32		.20		.15	
Sminthuridae			**.082**	NB	**.046**	NB	.57	NB	**.032**	
Thysanoptera			1.00	NB	.68					
Diptera (Trapped)			.44	L/P	.21					
Lepidoptera larvae			**.025**	NB		NB	**.008**	P	.61	
Larval Diptera			.76	Perm	**.031**					
Adult Diptera		**x**	**.014**	NB		P	.17	NB	**.039**	
Mixed Trophic Levels										
Larval Coleoptera			**.020**	NB		NB	**.093**	NB	.14	**Examine Plots; no consistent effect**
Adult Coleoptera			.87	NB	.39					
Predators										
Cursorial spiders			.16	L	.53					
All spiders (Kempson)			**.068**	Perm	.39	NB	.72	NB	**.015**	
Pseudoscorpiones			.77	NB	.22					
Web spiders			.78	L/NB	.69					
Chilopoda			1.00	Perm	.47					

Table S6.2: SUMMER, Years 2 and 3

Taxon (response variable)	Multivariate				Univariate							
	Spearman		Multiple		ALL PLOTS			Open		Fenced		
	Y2	Y3	Y2	Y3	R x Y x F [Model in ()]	R x Y	R	R x Y	R	R x Y	R	**Notes**
Fungivores/Detritivores												
Hypogastruidae	+	+	+	+	**.012** (Perm)			.32	.36	.32	.37	**Examine Plots; poor model behavior**
Onychuridae					.20 (Perm)	.18	.19					
Entomobryidae		+			.23 (NB)	.67	**.064**					
Isotomidae					.53 (Perm)	.64	.37					
Tomoceridae				–	.44 (Perm)	**.066**	**Plot Means across Years: Resource = . 11 [Yr2 = .91; Yr3 = .018]**					
Sminthuridae	+				.84 (P)	**.003**	**Year2, Resource = .006; Year3, Resource = .31**					
Thysanoptera					**.025** (NB)			.42	.12	**.004**	**.009**	
Diptera (Trapped)	+	+			.31 (NB)	.11	.11					**Examine Plots; marg. consistent effects**
Lepidoptera larvae					**.011** (P)			.45	**.10**	**.002**	.20	
Larval Diptera					.35 (L)	.29	.21					
Adult Diptera	+				.62 (NB)	.44	.13					
Mixed Trophic Levels												
Larval Coleoptera		+		+	**.077** (NB)	.69	**.017**	**.061**	.32	.61	**.002**	
Adult Coleoptera	+	+	+		.97 (P)	.73	.14					
Predators												
Cur spiders					.76 (Perm)	.81	.20					
All spiders (Kempson)			–		.81 (P)	.25	.46					
Pseudoscorpiones				–	**.009** (P)			.17(NB)	**.038**	.28	.51	**Examine Plots; complex pattern**
Web spiders					.50 (P)	**.091**	.77					
Chilopoda					.64 (Perm)	.73	.72					

Table S6.3: FALL, Year 1

Taxon (response variable)	Multivariate		Univariate							
			**ALL PLOTS**			**Open**		**Fenced**		
	Spearman	Multiple	R x F	Model	R	Model	R	Model	R	**Notes**
Fungivores/Detritivores										
Hypogastruridae			.64	NB	.41					
Onychuridae	+		.58	L/Tr	**.097**					**Linear Model, Square Root (y)**
Entomobryidae	+	+	.53	NB	**.008**					
Isotomidae	+		.96	NB	.68					
Tomoceridae	+		**.014**	NB		P	.15	NB	**.020**	
Sminthuridae	+		.62	NB	.90					
Thysanoptera			**.10**	Perm	**.046**	Perm	.70	Perm	.96	
Diptera (Trapped)			.38	P	.32					
Lepidoptera larvae			.55	NB	.44					
Larval Diptera			**.10**	Perm	**.092**	Perm	.28	Perm	.16	
Adult Diptera	+	+	.52	NB	.23					
Mixed Trophic Levels										
Larval Coleoptera			.39	NB	.21					
Adult Coleoptera			.33	NB	.28					
Predators										
Cursorial spiders	+		.14	NB	**.074**					
All spiders (Kempson)			.44	NB	.21					
Pseudoscorpiones			.25	NB	.71					
Web spiders	+		.91	NB	**.009**					
Chilopoda			**.091**	Perm	.36	Perm	**.047**	Perm	**.024**	**Examine Plots; complex pattern**

Table S6.4: FALL, Years 2 and 3

Taxon (response variable)	Multivariate				Univariate							
	Spearman		Multiple		**ALL PLOTS**			**Open**		**Fenced**		
	Y2	Y3	Y2	Y3	R x Y x F [Model in ()]	R x Y	R	R x Y	R	R x Y	R	**Notes**
Fungivores/Detritivores												
Hypogastruidae	+	+		+	.24 (Perm)	.24	**.007**	**Plot Means across Years: Resource = .001**				
Onychuridae	+		+		.98 (Perm)	**.02**	**.010**	**Year 2: Resource = .014; Year 3: Resource = .51**				
Entomobryidae	+				.22 (NB)	.13	**<.001**					
Isotomidae					.13 (Perm)	**.055**	**.060**	**Plot Means: Resource = .069 [Yr2 = .075; Yr3 = .053]**				
Tomoceridae					.90 (P)	.16	.76					
Sminthuridae	+		+		.81 (Perm)	**.003**	**.001**	**Year 2: Resource = .001; Year 3: Resource = .29**				
Thysanoptera	–				.75 (Perm)	.45	.53					
Diptera (Trapped)	+				.58 (P)	.26	**.097**					
Lepidoptera larvae		–			.75 (P)	.21	**.002**					
Larval Diptera	+	+			.26 (P)	.61	**<.001**					
Adult Diptera					.14 (Perm)	.42	.73					
Mixed Trophic Levels												
Larval Coleoptera	+				.14 (NB)	**.005**	**.004**					
Adult Coleoptera	+				.42 (NB)	**.001**	**.009**					
Predators												
Cursorial spiders					**.024** (NB)	.53	.15	**.005**	.33	.56	.18	
All spiders (Kempson)					.30 (P)	.52	.93					
Pseudoscorpiones					**.006** (NB)			.14	**.10**	**.003**	.17	**Examine Plots; complex pattern**
Web spiders		–		–	.70 (Perm)	.12	.58					
Chilopoda					**.087** (Perm)	1.00	.29	.25	.15	.27	.14	
