# Supplementary material for: Long-term resource addition to a detrital food web yields a pattern of responses more complex than pervasive bottom-up control
Source: PeerJ. 2017 Jul 21;5:e3572. doi: 10.7717/peerj.3572 (PMC5522720; doi:10.7717/peerj.3572)
Supplement: Appendix S7 [file peerj-05-3572-s007.pdf]

Supplemental **Appendix S7**

Arthropod Data Set

Each line in the accompanying comma-delimited file (Supplemental Data S8) represents a sample from one of the 20 experimental units.

Design Variables

Row	1-120; each row is a complete set of samples for one of
	20 experimental units for a single sampling period 7-28;
Plot	numbers used to designate each of 20 exp. units A =
Resource	Ambient, S = Supplemented
Fencing	F = Fenced, O = Open
Year	1, 2, 3 -- 1997, 1998, 1999, respectively
Season	S=Summer, F=Fall
	NOTE: Values for Year = 1, Season = S
	are averages of July and August samples

Response Variables

Kempson Samples	Number extracted per single 0.05 sq.-m sample of litter
Thy	Thysanoptera (thrips)
Acol	Beetles (Coleoptera) -- Adults
Adip	Diptera (flies) --- adults
Lcol	Beetle larvae
Llep	Lepidoptera (moths, etc) larvae
Ldip	Diptera larvae
Ara	Spiders (Araneae)
Pse	Pseudosorpiones(Pseudoscorpions)
Chi	Centipedes (Chilopoda)
Ent	Entomobryidae (Collembola --- springtails)
Iso	Isotomidae (Collembola --- springtails)
Tom	Tomoceridae (Collembola --- springtails) Onychiuridae
Ony	(Collembola --- springtails) Sminthuridae (Collembola
Smi	--- springtails) Hypogastruridae (Collembola ---
Hyp	springtails)
Sticky Trap Samples	Number per trap
TrpDip	Adult Diptera
Litter Sifting Samples	Number per 0.2 sq.-m sample of litter sorted in the field
Cur	Cursorial spiders
Web	Web-building spiders

No. of Response Variables = 18
